# In vivo structure-function studies of human hepatic lipase: the catalytic function rescues the lean phenotype of HL-deficient (*hl*^−/−^) mice

**DOI:** 10.14814/phy2.12365

**Published:** 2015-04-12

**Authors:** Jeffrey Chen, Karl J Kaiyala, Jennifer Lam, Nalini Agrawal, Lisa Nguyen, Kayoko Ogimoto, Dean Spencer, Gregory J Morton, Michael W Schwartz, Helén L Dichek

**Affiliations:** 1 Department of Pediatrics, University of WashingtonSeattle, Washington; 2 Department of Dental Public Health Sciences, School of Dentistry, University of WashingtonSeattle, Washington; 3 Department of Medicine, Diabetes and Obesity Center of Excellence, University of WashingtonSeattle, Washington

**Keywords:** Catalytic function, hepatic lipase, reduced energy expenditure, weight gain

## Abstract

The lean body weight phenotype of hepatic lipase (HL)–deficient mice (*hl*^−/−^) suggests that HL is required for normal weight gain, but the underlying mechanisms are unknown. HL plays a unique role in lipoprotein metabolism performing bridging as well as catalytic functions, either of which could participate in energy homeostasis. To determine if both the catalytic and bridging functions or the catalytic function alone are required for the effect of HL on body weight, we studied (*hl*^−/−^) mice that transgenically express physiologic levels of human (h)HL (with catalytic and bridging functions) or a catalytically-inactive (ci)HL variant (with bridging function only) in which the catalytic Serine 145 was mutated to Alanine. As expected, HL activity in postheparin plasma was restored to physiologic levels only in hHL-transgenic mice (*hl*^−/−^*hHL)*. During high-fat diet feeding, hHL-transgenic mice exhibited increased body weight gain and body adiposity relative to *hl*^−/−^*ciHL* mice. A similar, albeit less robust effect was observed in female hHL-transgenic relative to *hl*^−/−^*ciHL* mice. To delineate the basis for this effect, we determined cumulative food intake and measured energy expenditure using calorimetry. Interestingly, in both genders, food intake was 5–10% higher in *hl*^−/−^*hHL* mice relative to *hl*^−/−^*ciHL* controls. Similarly, energy expenditure was ∼10% lower in HL-transgenic mice after adjusting for differences in total body weight. Our results demonstrate that (1) the catalytic function of HL is required to rescue the lean body weight phenotype of *hl*^−/−^ mice; (2) this effect involves complementary changes in both sides of the energy balance equation; and (3) the bridging function alone is insufficient to rescue the lean phenotype of *hl*^−/−^*ciHL* mice.

## Introduction

Hepatic lipase (HL) is a multifunctional lipolytic enzyme expressed in the liver that plays a central role in lipoprotein metabolism (Olivecrona and Bengtsson-Olivecrona 1993; Connelly 1999; Brunzell and Deeb 2001). Hepatic lipase acts by catalyzing the hydrolysis of phospholipid and triglyceride in circulating lipoproteins remodeling them and reducing plasma lipid levels (Kuusi et al. 1979, 1980; Jackson 1983; Olivecrona and Bengtsson-Olivecrona 1993; Brunzell and Deeb 2001). For example, overexpression of human HL in rabbits and mice reduces plasma triglyceride and cholesterol levels (Fan et al. 1994; Dichek et al. 1998, 2004b). Hepatic lipase also acts by bridging lipoproteins onto cell surfaces in proximity to receptors for uptake (Ji et al. 1994, 1997). Once secreted, HL attaches to surfaces of hepatocytes and sinusoid endothelial cells via binding to heparan-sulfate proteoglycans (HSPG) (Jansen et al. 1979; Kounnas et al. 1995; Sanan et al. 1997; Dichek et al. 1998). HSPG binding is a critical component of the bridging function through which HL mediates cellular uptake of lipoproteins (Diard et al. 1994; Ji et al. 1997; Sendak et al. 2000). The binding of HL to HSPG requires interaction between negatively charged sulfate residues on the HSPG and positively charged amino acid residues in HL (van Tilbeurgh H et al. 1994; Sendak et al. 2000). This interaction can be disrupted by competitive binding of other positively charged molecules including heparin, as well as by removal of negatively charged sulfate groups by treatment with heparinase (Ji et al. 1997). Once bound to HSPG, the catalytic function of HL hydrolyzes lipoprotein remnants, intermediate density lipoproteins (IDL), low-density lipoproteins (LDL), and high-density lipoproteins (HDL), releasing-free fatty acids (FFA) and smaller lipoprotein particles (Dichek et al. 1998).

The contribution of HSPG-binding to the bridging function is illustrated by in vitro studies using McArdle Rat Hepatoma 7777cells (Ji et al. 1997). Those studies demonstrate that cellular binding and uptake of labeled lipoproteins increases when the cells are incubated with conditioned medium from cells transfected with either functional human HL or catalytically inactive HL (ciHL) (Ji et al. 1997). Thus, human HL can increase cell association and uptake of lipoproteins via the bridging function, even in the absence of its catalytic activity. Conversely, disruption of the bridging function by abolishing HSPG binding using heparinase decreases cell-association of lipoproteins (Ji et al. 1997). Also, in in vivo studies, intraportal injection of heparin blocks the physical association of HL with hepatocytes in mice with liver-directed transgenic expression of human HL, confirming the importance of the bridging function of HL (Dichek et al. 1998). Thus, the catalytic and bridging functions of HL are complementary, and the impact of HL on lipoprotein metabolism depends upon both.

Previous work examining the respective roles of human HL's catalytic and bridging functions in lipoprotein metabolism and atherosclerosis unexpectedly revealed reduced weight gain in mice expressing supraphysiologic levels of ciHL (Dichek et al. 2004b). Also, recent work suggests that mice lacking HL are leaner than normal controls, especially when fed an obesity-promoting high-fat diet (HFD) (Chiu et al. 2010). Although the underlying mechanisms remain unknown, the effect involves both reduced food intake and a relative increase in energy expenditure, irrespective of diet (Chiu et al. 2010). Also unknown is whether the catalytic or bridging functions (or both) mediate HL's effects on energy homeostasis and obesity susceptibility (Dichek et al. 1998, 2001, 2004a). To address this question, we rescued mice lacking HL with transgenic expression of the cDNA encoding either intact human HL (possessing both its catalytic and bridging functions) or a catalytically inactive HL ((ci)HL) mutant (possessing its bridging function only). The resultant mice express physiologic HL levels and were used to determine (1) whether the catalytic function of HL is required to rescue the body weight phenotype of *hl*^−/−^ mice, or (2) if the bridging function alone is sufficient to mediate this effect.

## Materials and Methods

### Genetically engineered mice

HL- deficient mice on the C57BL/6 background (*hl*^−/−^) (Homanics et al. 1995) (http://jaxmice.jax.org/strain/002056.html) were obtained from our colony at the University of Washington as described (Chiu et al. 2010). The human HL- and ciHLcDNAs used to produce the humanized mouse models were reported previously (Ji et al. 1997). For comparison, data from *WT* (*hl*^+/+^) C57BL/6 mice from our earlier study are included (Chiu et al. 2010).

### Human HL transgenic mouse model

Transgenic mice that express physiologic levels of human HL were generated by the Transgenic Resources Program at the University of Washington using a construct containing sequences from the human apoE gene locus, including the hepatic control region that directs transgene expression to the liver (a gift from Dr. John Taylor at the Gladstone Institutes of Cardiovascular Disease) (Dichek et al. 1998) (Fan et al. 1994).

The human HL transgenic founder was 75% C57BL/6/25% C3H background and was bred with *hl*^−/−^ mice on the C57BL/6 background. The resulting transgenic offspring was backcrossed with *hl*^−/−^ to achieve at least 94% C57BL/6 background.

### Human ciHL gene targeted model

Mice expressing human catalytically inactive (ci) HL (Ji et al. 1997) at physiologic levels were generated by homologous recombination in C57BL/6 embryonic stem cells in collaboration with Genoway (Lyon, France). Mouse HL sequences were amplified from an HL-specific BAC clone derived from a BAC library of the C57BL/6 mouse strain (Wellcome Trust Sanger Institute, Hinxton, Cambridgeshire, UK). The targeting vector features the Neomycin-positive selection cassette flanked by loxP sites, a 6.1 kb long homology region, a 1.6 kb short homology region, and the 1.6 kb ciHL cDNA (Ji et al. 1997). The targeting vector disrupts the exon 2 coding sequence in the mouse HL gene by in-frame fusion of the human ciHLcDNA with the murine ATG transcriptional start site creating a humanized knock-in allele. Sequence confirmation was performed by restriction digests and DNA sequencing throughout the vector construction. The targeting vector was linearized by restriction digest and transfected into C57BL/6 ES cells by electroporation. Successful homologous recombination at the 5′ end of the mouse HL locus was detected by PCR and confirmed by Southern blot analysis, which verified the 3′ homologous recombination event in three clones that were positive for the 5′ end screen. Southern blot hybridization confirmed the absence of randomly integrated copies of the targeting construct. Embryonic stem cell clones demonstrating successful homologous recombination were injected into blastocysts isolated from albino C57BL/6 (C57BL/6J-Tyrc-2J/J) female mice. The injected blastocysts were reimplanted into OF-1 pseudopregnant females for generation of chimeric offspring. Male offspring with >50% chimerism were bred with C57BL/6 Cre recombinase-expressing deleter mice to excise the neomycin selection cassette. Offspring with black coat color (heterozygous for the knock-in allele) were analyzed by PCR to verify excision of the neomycin selection cassette and to confirm the absence of the mouse HL (Table1). Presence of the knock-in and wild-type alleles was verified by Southern blot analysis. Mice that were heterozygous for the knock-in allele were mated with each other to produce homozygous knock-in mice. Male and female mice were studied. Mice were housed in a modified barrier facility with a 12-h light, 12-h dark cycle. All experiments were approved by the Institutional Animal Care and Use Committee and the Office of Animal Welfare of the University of Washington.

**Table 1:** Primer sequences to detect mouse HL and human ciHL.

Name	Sequence	Purpose
Forward 57533hom	5′-ATG CGA CTA GAA AGA CCA GGA CCA CG-3′	Detects mouse HL, 977 bp, In WT and heterozygous mice
Reverse 57534hom	5′-AAG GCG ATT TCA CAA CCC CAA TAG G-3′	Detects mouse HL, 977 bp, In WT and heterozygous mice
Forward 57545bct	5′-AGG GTT ACA TCA CAC CAC CCA TCG TC-3′	Detects the ciHL Cre-excised allele, 1683 bp
Reverse 57546bct	5′-GAG AAA CAC AGG GGA CTT GTG TCC ATG-3′	Detects the ciHL Cre-excised allele, 1683 bp

### Expression of human HL and ciHL

Presence of the hHL and ciHL transgenes was confirmed in vivo by PCR that specifically detects human HL (Dichek et al. 2004a). Hepatic lipase activities in plasma obtained 10 min after tail vein injection of heparin (150 U/kg body weight) were determined as previously described with [1-^14^C] trioleate-labeled emulsion (Iverius and Brunzell 1985). Specifically, hepatic lipase activities were obtained after suppressing lipoprotein lipase activity with 1 mol/L sodium chloride (Iverius and Brunzell 1985; Nilsson-Ehle 1987). HL protein expression was confirmed by Western blot analysis using a monospecific polyclonal rabbit anti-human HL antiserum as described (Dichek et al. 1998, 2001).

### Diet study

Groups of male and female mice were weaned, caged individually and fed a HFD containing 42% of calories from fat and 0.15% (wt/wt) cholesterol (TD 88137, Harlan Teklad, Madison, WI) starting at 6–8 weeks of age. Food intake and body weight were measured daily between 9 and 11 am for 3 months. Body lengths were measured in adult mice (24–26 weeks old).

### Adipocyte histology

Gonadal white adipose tissue (WAT) was fixed in 10% Neutral Formalin and embedded in paraffin. For each tissue sample, 5 *μ*m thick sections were cut and stained by hematoxylin and eosin. Noncontiguous sections were visualized using an Olympus BH-2 microscope and photographed using an Olympus DP-72 camera at 20× magnification. The area of image was 250,855.5 *μ*m^2^. Between 148 and 295 cells were counted per tissue by two blinded observers. The correlation between observers was 0.94. The area of the section was divided by the mean of the cell count from each observer and expressed as adipocyte size in *μ*m^2^.

### Body composition and Calorimetry

Body composition was measured at age 22–24 weeks in the Energy Balance Glucose Metabolism Core (EBGM) of the NIH-funded Nutrition Obesity Research Center (NORC) at the University of Washington (depts.washington.edu/uwnorc/) using the EchoMRI^tm^ 3-in-1 Animal Tissue Analyzer (Echo Medical Systems, Houston, TX). Energy Expenditure, Respiratory Quotient, and Ambulatory Activity level were determined continuously over 36 h by indirect calorimetry and food intake continuously monitored using the Comprehensive lab Animal Monitoring System (Columbus Instruments Co., Columbus, OH) also located within the EBGM Core as previously described (Gelling et al. 2008; Morton et al. 2011). To control for the influence of body size variation on total energy expenditure, group comparisons involving this outcome were adjusted for total body mass using analysis of covariance (ANCOVA), as recommended (Kaiyala et al. 2010; Kaiyala and Schwartz 2011).

### Plasma measurements

Enzyme-linked immunosorbent assays (ELISA) were used to measure leptin (Crystal Chem, Inc, Downer's Grove, IL) and insulin (ALPCO, Salem, NH). Blood glucose was measured using Truetest glucose test strips and glucometer (Nipro diagnostics, Osaka, Japan).

Plasma lipoproteins were separated by fast protein liquid chromatography (FPLC) and cholesterol concentrations were determined by a standard colorimetric assay as described (Qian et al. 2007). All samples were from mice fasted for 4–6 h except for glucose in which animals were fasted for 16 h.

### Measures of glucose homeostasis

At the end of the diet intervention, intraperitoneal glucose tolerance testing (IPGTT) was performed in the NIH-funded Mouse Metabolic Phenotyping Center of the University of Washington. Groups of mice (*n* = 4/group) from each of the 3 genotypes (*hl*^−/−^, *hl*^−/−^ *hHL* and *hl*^−/−^ *ciHL*) were fasted for 16 h (overnight) and subsequently received a glucose bolus (1 g/kg ip). Blood glucose levels were measured at 0, 15, 30, 60, and 120 min and area under the curve analyses of the integrated glucose response to the IPGTT were performed using the trapezoidal rule (Purves 1992).

### Measurement of adrenal stress response

In a separate study, *WT, hl*^−/−^ *hHL* and *hl*^−/−^ *ciHL* mice on HFD were subjected to a 16 h fast and blood withdrawn with minimal handling (<1 min) for duplicate corticosterone measurements, using the Mouse/Rat corticosterone ELISA 55-CORMS-E01 kit, ALPCO Diagnostics (Salem, NH).

### Statistical analyses

Results are expressed as mean ± SD unless specified otherwise. Student's t-test for independent samples was employed for two group analyses and one-way analysis of variance (ANOVA) was used for three group comparisons using Statview (SAS Institute, Cary, NC). Analysis of covariance (ANCOVA) was used on pooled data from male and female mice to compare energy expenditure between genotypes after adjusting for differences in body mass and composition. Statistical analyses of energy expenditure were performed by the NORC Biostatistics Subcore using SPSS (version 19, IBM Corp., Somers, NY). No statistical analysis was performed for the historical data on *WT* mice.

## Results

### Expression of hHL and ciHL

**Figure 1:**
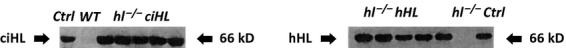
Western blot analysis for human HL immunoreactivity. Expression of human HL in postheparin plasma from *hl*^−/−^ *ciHL* (Left panel) and *hl*^−/−^ *hHL* mice (Right panel) were assayed by Western blot using a monospecific antihuman HL polyclonal antibody (12). As expected immunoreactivity was present in plasma from ciHL- and hHL- expressing mice and absent in negative control *WT* (with murine HL) and *hl*^−/−^ mice. Positive controls were plasma from *hl*^−/−^ *ciHL* mice (Left panel) and *hl*^−/−^ *hHL* (Right panel).

Expression of active and inactive human HL in vivo was confirmed by analysis of postheparin plasma and demonstrated physiologic HL activity (in the range of human HL activity) in *hl*^−/−^ hHL mice but not in either *hl*^−/−^ mice that lack HL or in *hl*^−/−^ *ciHL* mice rescued with the catalytically inactive transgene (Table2). As expected, HL activity was present in preheparin plasma from *WT* mice, reflecting the reduced heparin affinity of murine HL that allows a large portion of HL to circulate (Table2). Background activity from incompletely suppressed lipoprotein lipase was detected in pre-heparin plasma from *hl*^−/−^, *hl*^−/−^ *ciHL*, and *hl*^−/−^ *hHL* mice as well as in postheparin plasma from *hl*^−/−^ and *hl*^−/−^ *ciHL* mice (Table2). Western blot analysis confirmed the presence of immunoreactive human HL in both *hl*^−/−^ *hHL* and *hl*^−/−^ *ciHL* mice and its absence in *hl*^−/−^ and *WT* mice (Fig. 1).

**Table 2:** Hepatic lipase activities in pre and postheparin plasma^1^.

Genotype	Male		Female	
	Preheparin^2^	Postheparin^3^	Preheparin^4^	Postheparin^5^
WT (*hl*^+/+^)	5.0 ± 0.3	9.0 ± 0.2	5.7 ± 0.5	8.7 ± 0.4
*hl*^−/−^	1.1 ± 0.3	2.1 ± 0.5	0.7 ± 0.1	1.2 ± 0.1
*hl*^−/−^ hHL	0.9 ± 0.2	18.9 ± 3.3	0.9 ± 0.1	16.2 ± 4.1
*hl*^−/−^ciHL	1.7	1.8 ± 0.3	1.5 ± 0.3	1.2 ± 0.1
Homo sapiens^6^	NA	13.3 ± 5.7	NA	7.1 ± 3.2

1 HL activity measured as *μ*Eq FFA/mL/h.

2 *n* = 3 except *hl*^−/−^ciHL, *n* = 1.

3 *n* = 4 except WT, *n* = 3.

4 *n* = 3.

5 *n* = 4 except WT, *n* = 3.

6 From ref. (Carr et al. 2001); NA, not available.

### Genotype differences in body weight and body fat mass

Body weight at baseline was similar among the three genotypes, HL-deficient mice alone or expressing either intact hHL or catalytically inactive ciHL (Table3). However, following exposure to HFD for three months, male *hl*^−/−^ mice rescued with transgenic expression of intact hHL (possessing both the catalytic and bridging functions) had significantly higher body weight (∼15%) than either male mice rescued with inactive ciHL (which contains the bridging function, but is catalytically inactive) or *hl*^−/−^ male mice alone (*P* < 0.02 for both). In contrast, there was no difference in body weight between *hl*^−/−^ mice and those rescued with the inactive ciHL (*P* = ns). A similar pattern was seen with body weight gain over the course of the study, such that male *hl*^−/−^ mice rescued with the human active HL gained more weight on the HFD than either *hl*^−/−^ mice alone or those mice rescued with ciHL (*P* < 0.05 for both) (Table3).

**Table 3:** Physical characteristics, food intake and metabolic responses to High-fat diet challenge.

	Males			Females			Female Historical data^15^
	*hl*^−/−^hHL (8)	*hl*^−/−^ (6)	*hl*^−/−^ciHL (13)	*hl*^−/−^hHL (8)	*hl*^−/−^ (8)	*hl*^−/−^ciHL (11)	WT(*hl*^+/+^) (5)
Weight (g) Baseline	23.7 ± 1.1	23.9 ± 0.6	23.3 ± 1.6	19.5 ± 0.8	19.3 ± 1.0	19.1 ± 4.4	19.0 ± 1.3
Weight (g) End	37.2 ± 4. 1^1^	33.1 ± 3.5	31.9 ± 3.7	26.5 ± 2.5^2^	25.3 ± 2.4	23.7 ± 2.1	28.7 ± 4.1
Delta weight (g)	13.5 ± 4.4^3^	9.1 ± 3.2	9.4 ± 3.5	7.0 ± 2.6^4^	5.5 ± 2.2	4.6 ± 1.6	9.7 ± 3.0
Body fat (%)	39.7 ± 6.0	32.8 ± 8.0	32.8 ± 8.0^5^	31.3 ± 5.5^6^	29.8 ± 6^6^	26.7 ± 7.9^7^	34.0 ± 6.1
Length (cm)	10.3 ± 0.2	10.4 ± 0.2	10.1 ± 0.2^5^	9.9 ± 0.1	9.9 ± 0.1^8^	9.8 ± 0.1^9^	10.6 ± 0.2
Cumulative Food Intake (Kcal)	1099 ± 89^7^	1066 ± 73	1019 ± 88	1041 ± 51^8^^,^^10^	982 ± 13^5^	949 ± 72	1124 ± 135
Leptin (ng/mL)	52.3 ± 24.6^5^	21.4 ± 12.3^11^	43.3 ± 8.4^8^	47.1 ± 21.4^5^	37.1 ± 3.7^7^	18 ± 11.6 12^,^^13^	NA
Insulin (ng/mL)	2.3 ± 1.4	1.5 ± 0.8	1.7 ± 0.7^8^	0.7 ± 0.4^5^	0.7 ± 0.4^5^	1.1 ± 0.7^12^	NA
Glucose (mg/dL)	207 ± 34^7^^,^^14^	161 ± 25^7^	143 ± 23^8^	167 ± 24^7^	158 ± 20^7^	151 ± 23^6^	NA

1 *P* < 0.02 vs. male *hl*^−/−^ciHLmice.

2 *P* = 0.05 vs. female *hl*^−/−^ciHL mice.

3 *P* < 0.05 vs. male *hl*^−/−^ciHL and *hl*^−/−^mice.

4 *P* = 0.07 vs. female *hl*^−/−^ciHL mice.

5 *n* = 5.

6 *n* = 7.

7 *n* = 4.

8 *n* = 6.

9 *n* = 9.

10 *P* = 0.02 vs. female *hl*^−/−^ciHL mice.

11 *n* = 3.

12 *n* = 8.

13 *P* < 0.01 vs. female *hl*^−/−^hHL female mice.

14 *P* = 0.01 vs. male *hl*^−/−^ciHL, and *hl*^−/−^mice.

15 from ref (Chiu et al. 2010), NA, not available.

**Figure 2:**
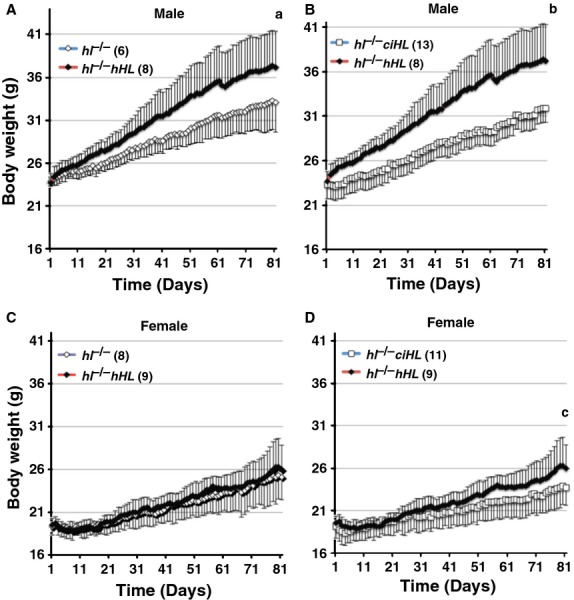
The catalytic function of human HL rescues the lean phenotype of HL-deficient mice. HF-fed *hl*^−/−^ *hHL* (filled diamonds), *hl*^−/−^ open diamonds) and *hl*^−/−^ *ciHL* (open squares) male (A, B) and female (C, D) mice. *hl*^−/−^ *hHL*, *hl*^−/−^ and *hl*^−/−^ *ciHL* mice were fed a HF-diet (42% of calories from fat) and weighed daily for 3 months. Group differences in body weight on day 81 were analyzed by One-way ANOVA: a, *P* < 0.02 vs*. hl*^−/−^ *hHL* males; b, *P* < 0.02 vs*. hl*^−/−^ *hHL* males; c, *P* = 0.05 vs*. hl*^−/−^ *hHL* females. Results are shown as mean ± SD.

In female *hl*^−/−^ mice, rescue with transgenic expression of intact hHL also resulted in significantly increased body weight (11%) relative to those rescued with inactive, ciHL (*P* = 0.05) (Table2 and Fig.2). In addition, there was a trend toward increased body weight gain in *hl*^−/−^ mice rescued with intact hHL relative to mice rescued with the inactive ciHL (Table3). Historical data from female *WT* mice with intact murine HL activity demonstrated a similar weight gain to the hHL-expressing mice (Table3) (Chiu et al. 2010). Taken together, these findings demonstrate that the HL catalytic function rescues the lean phenotype of *hl*^−/−^ mice although this effect is more pronounced in male than in female mice.

To determine whether these differences in body weight were due to changes in lean mass, fat mass or both, we performed body composition analysis. The increased body weight in HFD-fed male *hl*^−/−^ mice rescued with intact HL was characterized by an increase in body adiposity relative to either *hl*^−/−^ mice or *hl*^−/−^ mice-expressing ciHL (Table3). In female mice a similar but weaker trend was seen (Table3). In contrast, body length of either gender did not differ between genotypes, indicating that changes of body weight and fat mass are not due to alterations in linear growth (Table3). The length of WT mice from our previous study was comparable to the *hl*^−/−^ female mice in that study.

### Adipocyte histology

**Figure 3:**
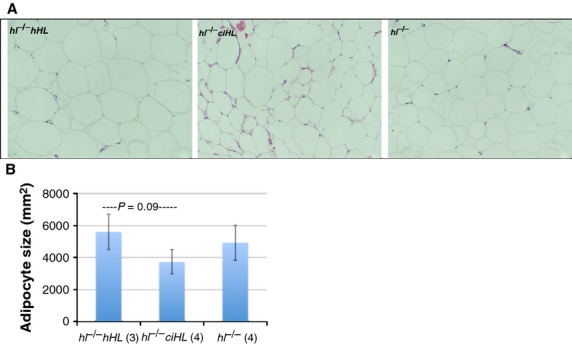
Histology of white adipose tissue. White adipose tissue characteristics in high-fat diet fed *hl*^−/−^ *hHL* (Top Left), *hl*^−/−^ *ciHL* (Top Center) and *hl*^−/−^ male mice (Top Right). (A) Hematoxylin and Eosin-stained gonadal tissue. (B) Quantitation of adipocyte size. Note trend toward increased size of adipocytes in *hl*^−/−^ *hHL* vs*. hl*^−/−^ *ciHL* mice.Values are shown as mean ±SD.

Using histochemical analysis, we further revealed that adipocytes from WAT of male *hl*^−/−^ *hHL* were 33% larger than from WAT of *hl*^−/−^ *ciHL* mice (5599 ± 1116 *μ*m^2^ vs. 3726 ± 1105 *μ*m^2^; *P* = 0.09), indicating that the increase in total fat mass induced by HL rescue is accompanied by a trend toward a proportional increase in adipocyte size (Fig.3). In our previous study, fat pads were 53% larger in WT when compared to *hl*^−/−^mice.

Together, these results suggest (1) that the resistance to diet-induced obesity (DIO) conferred by HL deficiency depends critically on absence of the enzyme's catalytic function; and (*2*) transgenic expression of an HL mutant with intact bridging function is insufficient to reverse the lean phenotype of mice lacking HL.

### Genotype differences in food intake, energy expenditure, RQ, and activity

We next determined whether these genotype differences in weight gain and body composition were due to changes of food intake, energy expenditure, or both. Cumulative food intake over the entire period of HFD feeding was increased by 10% in female *hl*^−/−^ mice-expressing active HL compared with those expressing the ciHL mutant (*P* = 0.02), and a similar trend was observed in male *hl*^−/−^ mice rescued with intact human HL (*hl*^−/−^ *hHL*) compared to those expressing ciHL, although the difference did not reach statistical significance (Table3). These data suggest that increased food intake contributed to the greater weight gain of HL-deficient mice rescued with transgenic expression of human HL consistent with the increased food intake in WT mice in our previous study (Table3).

**Figure 4:**
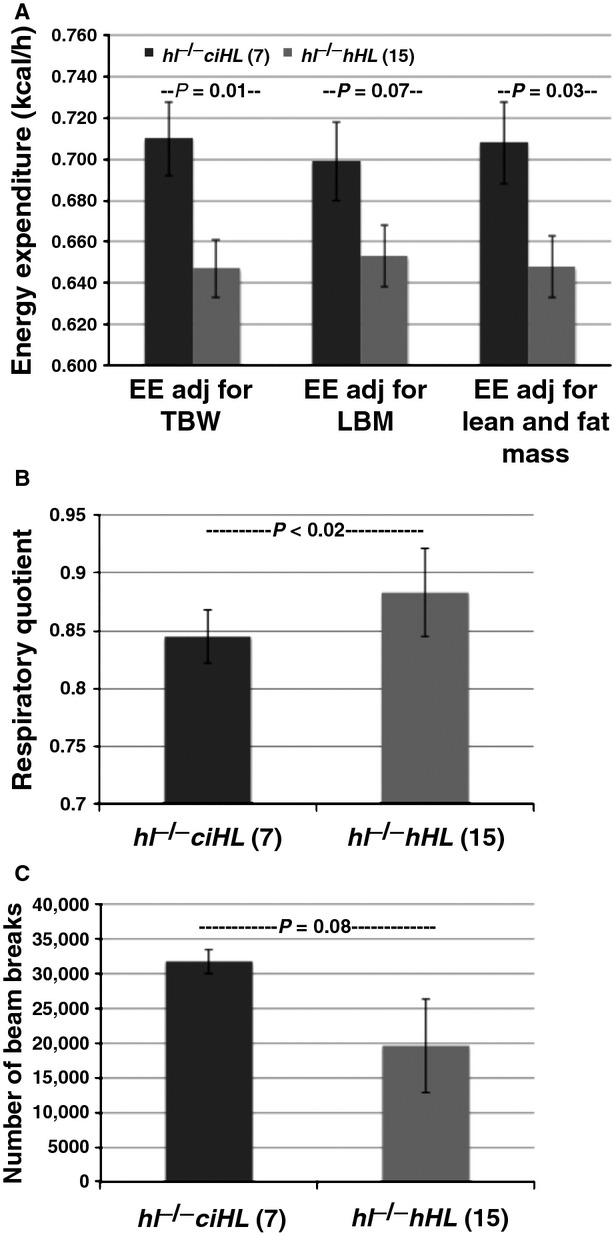
*hl*^−/−^ *ciHL* mice have increased adjusted expenditure (EE). (A) Energy expenditure (male and female mice combined) adjusted using ANCOVA for total body weight (TBW), lean body mass (LBM), and lean and fat mass. (B) Respiratory quotient (RQ) and (C) ambulatory activity in *hl*^−/−^ *ciHL* relative to *hl*^−/−^ *hHL* mice. Values are shown as mean ± SD.

Using indirect calorimetry, we found that energy expenditure adjusted for total body mass was decreased by 9% for a mean reduction of 0.064 ± 0.023 kcal/h in HL-deficient mice expressing active HL compared to mice expressing the inactive ciHL mutant (*P* = 0.012). A similar result was obtained when energy expenditure was adjusted for both lean and fat mass, which revealed a mean reduction of 0.060 ± 0.026 kcal/h (*P* = 0.03) in HL-deficient mice expressing active HL (Table4 and illustrated in Fig.4A).

**Table 4:** Energy expenditure in mice with active and inactive human hepatic lipase.

	*hl*^−/−^ ciHL (9)	*hl*^−/−^ hHL (15)	Mean difference
EE adjusted for TBW (kcal/h)^3^	0.710 ± 0.018	0.647 ± 0.014	0.064 ± 0.023^4^
EE adj for LBM (kcal/h)	0.699 ± 0.019	0.653 ± 0.015	0.046 ± .024
EE adj for lean and fat mass (kcal/h)	0.708 ± 0.020	0.648 ± 0.015	0.060 ± 0.026^5^

TBW, total body weight; LBM, lean body mass; EE, energy expenditure;

Values are shown as mean ± SE.

3 Average bodyweight 32.8542 g;

4 *P* = 0.01.

5 *P* = 0.03.

In addition, we found that RQ was significantly increased in HL-deficient mice expressing active HL compared to mice expressing ciHL: RQ 0.883 ± 0.038 for *hl*^−/−^ *hHL* mice vs. 0.845 ± 0.023 for *hl*^−/−^ *ciHL* mice, *P* = 0.01 (Fig.4B). These results suggest that *hl*^−/−^ *ciHL* mice (lacking the catalytic function) preferentially burn calories from fat, consistent with their lean phenotype.

We also found a trend toward reduced ambulatory activity levels in HL-deficient mice expressing active HL compared with mice expressing inactive ciHL, with the number of beam breaks being nonsignificantly lower in *hl*^−/−^ *hHL* than *hl*^−/−^ *ciHL* mice (19,607 ± 6747 vs. 31,743 ± 17,491; *P* = 0.076) (Fig.4C).

These results confirm previous evidence that HL deficiency increases energy expenditure and fat oxidation in mice, and demonstrates that the enzyme's catalytic function is required to reverse these effects.

### Genotype differences in plasma variables and glucose homeostasis

Because plasma leptin levels reflect the degree of adiposity, we postulated that values would be reduced in lean *hl*^−/−^ mice and that this effect would be reversed by transgenic expression of intact HL, but not the ciHL mutant. As expected, leptin levels were highest in mice with active HL and decreased in both genotypes of mice that lacked HL catalytic activity. Specifically, leptin levels in female *hl*^−/−^ *hHL* mice on the HFD were 47.1 ± 22 ng/mL compared to 37.1 ± 3.7 ng/mL and 18.0 ± 11.6 ng/mL in *hl*^−/−^ and *hl*^−/−^ *ciHL* mice, respectively (*n* = 4–8/group, *P* < 0.01) (Table3). In male mice, a similar trend was observed, although differences did not achieve statistical significance.

Surprisingly, although plasma insulin levels were higher in male than female mice (as expected) there were no genotype differences despite substantial differences in body weight and fat mass (Table3). Nevertheless, plasma glucose levels were considerably higher in male mice expressing intact human HL (207 ± 34 mg/dL) compared to either *hl*^−/−^ *ciHL* mice (143 ± 23 mg/dL), or *hl*^−/−^ (161 ± 25 mg/dL) (*P* = 0.01 for both comparisons). In contrast, plasma glucose levels did not differ between genotypes in female mice (Table3).

**Figure 5:**
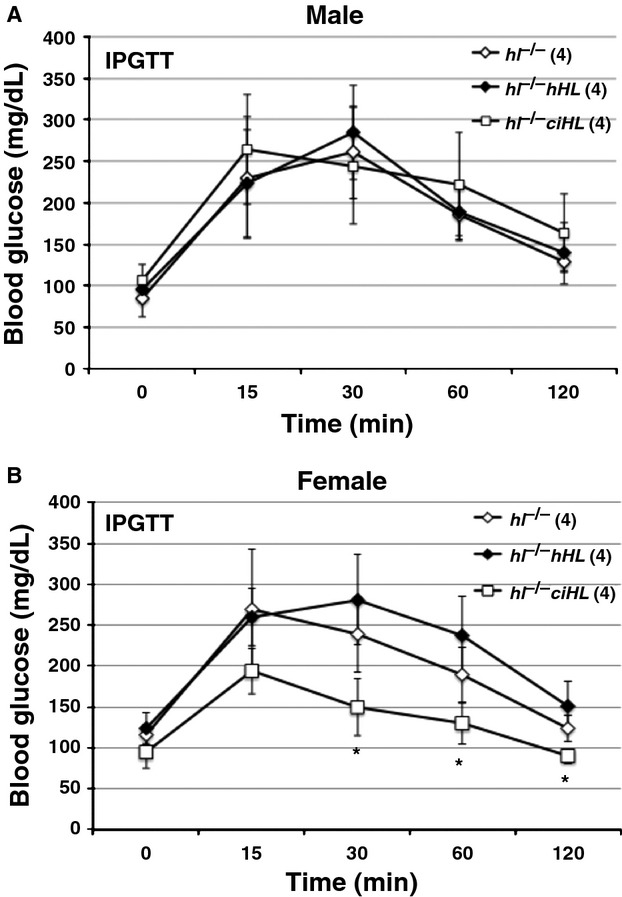
Effect of rescue of hepatic lipase deficiency on glucose tolerance. *Intraperitoneal glucose tolerance test* (IPGTT) in HF-fed *hl*^−/−^, *hl*^−/−^ *hHL* and *hl*^−/−^ *ciHL* in male (A) and female (B) mice. Mice were fasted overnight and received 1 g/kg glucose by i.p. inj. *P <* *0.05. Values are shown as mean ± SD.

To explore the glucose metabolic phenotype further, we challenged the mice with an intraperitoneal glucose tolerance test (ipgtt). Surprisingly, despite distinct differences in weight and fat mass in male mice the ipgtt was not different between the three genotypes (Fig.5A), and area under the curve (AUC) analyses of blood glucoses of all three genotypes showed no difference in glucose tolerance [AUC: *hl*^−/−^ *HL* 425 ± 75 (*n* = 4), *hl*^−/−^ *ciHL* 418 ± 99 (*n* = 4), and *hl*^−/−^433 ± 59 (*n* = 4), *P* = NS]. Therefore, in male mice, despite distinct differences in weight and fat mass, there was no consistent difference in glucose tolerance between genotypes.

In contrast, in female mice, those with active HL displayed increased plasma glucose levels at 30, 60 and 120 min postglucose challenge (Fig.5B), and increased AUC analyses of blood glucoses: AUC: *hl*^−/−^ *HL* 449 ± 65 (*n* = 4), *hl*^−/−^ *ciHL* 259 ± 41(*n* = 4), and *hl*^−/−^ 376 ± 64 (*n* = 4), *P* < 0.005, compared with catalytically inactive ciHL-expressing mice. Therefore, in females the differences in weight and fat mass was accompanied by a modest difference in glucose tolerance between genotypes.

### Plasma lipoprotein cholesterol profiles

**Figure 6:**
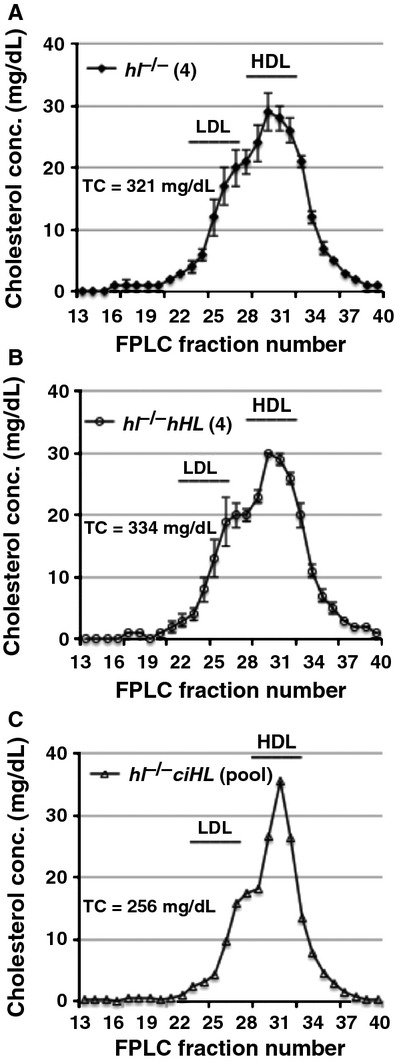
FPLC profiles of plasma cholesterol from fasted, high- fatfed male mice. (A) *hl*^−/−^ (n = 4), (B) *hl*^−/−^ hHL (n = 4), and (C) hl^−/−^*ciHL* (pool of three mice). Plasma was fractionated by Superose 6 chromatography and fractions assayed for cholesterol. The distribution of LDL and HDL are indicated by horizontal bars. (A andB), Tracings represent the average ± SD of four individual FPLCs.(C), Tracing of sample pooled from three mice.

We previously demonstrated that supraphysiologic levels of ciHL and hHL reduces the elevated plasma cholesterol levels in HFD fed mice on the *hl*^−/−^ genetic background. There was minimal to no effect on triglyceride levels in those studies. We speculated that much lower, physiologic levels of ciHL and hHL would have minor effects on the plasma cholesterol and indeed this speculation was borne out by our results. Lipoprotein cholesterol profiles were comparable among the *hl*^−/−^, *hl*^−/−^ *hHL*, and *hl*^−/−^ *ciHL* genotypes (Fig.6A–C).

### Adrenal corticosterone response to stress

According to previous data in mice, HL is present in the adrenal gland and HL activity may be required for a normal adrenal stress response. Also, in mice, HL deficiency is associated with a decreased stress response. In turn, a decreased adrenal stress response (adrenal insufficiency) leads to reduced weight gain. We hypothesized that the lack of rescue of the lean phenotype in *hl*^−/−^ mice by expression of ciHL, was due to reduced adrenal stress response (because of absent HL activity). To assess the adrenal stress response we measured plasma corticosterone levels in *WT, hl*^−/−^ *hHL, and hl*^−/−^ *ciHL* mice in response to a prolonged fast. We used plasma corticosterone levels in the fed state as a control. Contrary to our hypothesis, all three genotypes demonstrated similar corticosterone levels in response to the stress of fasting: [*WT, 135 ± 53 *ng/mL (*n* = 4)*, hl*^−/−^ *hHL, 111 ± 41 *ng/mL (*n* = 6)*, and hl*^−/−^ *ciHL,* 136 ± 77 ng/mL (*n* = 4), *P* = NS]. Simultaneously obtained baseline corticosterone levels in a cohort of fed mice were [*WT 13 ± 7 *ng/mL (*n* = 4)*, hl*^−/−^ *hHL, 36 ± 33 *ng/mL (*n* = 5*), and hl*^−/−^ *ciHL,* 35 ± 32 ng/mL (*n* = 3), *P* = ns]. Based on these results, it is unlikely that the lack of rescue of the lean phenotype in *hl*^−/−^ mice is caused by a reduced adrenal stress response.

## Discussion

Previous evidence suggests that mice deficient in HL exhibit a lean phenotype and are protected against DIO. Here, our findings demonstrate that the catalytic function of HL is required for HFD-induced weight gain. Specifically, rescue of HL-deficient mice with transgenic expression of the intact human HL (possessing both its catalytic and bridging functions) reverses the lean phenotype characteristic of HL-deficiency, and this effect was not observed when rescue was attempted with the catalytically inactive HL mutant. Moreover, this rescue of body weight and body fat gain was due to a combination of both reduced energy expenditure and modestly increased food intake. Taken together, these results highlight a novel anabolic role for HL's catalytic function to promote positive energy balance during HFD feeding.

Existing data in mice and humans support a role for HL in energy metabolism and weight regulation. In mice, genetic studies indicate that quantitative trait loci on chromosome 2, 5, and 7 are coincident for HL activity, percent body fat, and plasma cholesterol (Warden et al. 1995; Mehrabian et al. 1998). In humans, two separate studies of premenopausal women found that increased HL activity was correlated with increased intra-abdominal fat (assessed by computed axial tomography) (Despres et al. 1989; Carr et al. 1999) supporting our current findings of increased weight gain in mice with active human HL. Conversely, decreased intra-abdominal fat was correlated with reduced HL activity in a study designed to examine the effect of weight loss on plasma lipids (Purnell et al. 2000) supporting our current findings of reduced weight gain in mice without active human HL. In addition, our previous work showed that mice lacking HL have a lean phenotype with reduced weight gain on a HFD (Chiu et al. 2010), and our new data extend this work by showing that this phenotype is reversed when *hl*^−/−^ mice are rescued with transgenic expression of fully functional human HL, but not when they are rescued with a mutant HL that lacks catalytic function. Since the bridging function of HL is intact in both genotypes, this observation implies that HL bridging to the cell surface does not impact energy homeostasis when the catalytic function is absent. Thus, the catalytic function of HL is required for normal weight gain in mice fed a HFD.

Interestingly, the role of HSPG binding as sole mediator of the bridging function of the related enzyme, lipoprotein lipase (LPL) has come into question (Beigneux et al. 2007). Thus, a new accessory protein has been identified for LPL, glycosylphosphatidylinositol-anchored high-density lipoprotein-binding protein 1(GPIHBP1) (Beigneux et al. 2007; Davies et al. 2010). This protein transports LPL from the subendothelial space across the endothelial cell to its binding sites on the lumenal surface (Beigneux et al. 2007; Davies et al. 2010). It is possible that a protein similar to GPIHBP1 exists for hepatic lipase to enable its transport to the lumenal surfaces of endothelial cells, however such a protein remains to be discovered.

A noteworthy distinction between the biology of murine and human HL is that the latter is largely bound within the liver whereas the former is found largely in plasma (Peterson et al. 1986; Dichek et al. 1998). The localization of HL therefore differs between normal mice and HL-deficient mice rescued with transgenic expression of human HL. Although the basis for this difference is incompletely understood, it likely reflects differences in the bridging function that allows most HL in mice, but not humans, to circulate in proximity to lipoproteins. In this way, differences in the bridging function may influence the access of HL to lipoprotein substrates for both hydrolysis and for uptake. Combined with our findings that (1) transgenic expression of human HL appears to reverse the body weight phenotype of *hl*^−/−^ mice and that the catalytic function is required for this effect, and (2) expression of a HL mutant with intact bridging (but not catalytic) function does not rescue the body weight phenotype of *hl*^−/−^ mice, our data imply that the bridging function (which determines whether HL is localized to the liver or circulates in plasma) is not a key determinant of its effects on energy homeostasis.

This being said, our previous study revealed that during HFD feeding, the increase in percent body fat in *WT* mice (with murine HL) relative to *hl*^−/−^ mice (32%) (Chiu et al. 2010) was greater than our current finding in *hl*^−/−^ *hHL* mice (17%). The more modest increase in body fat content of mice with human- compared with murine HL may therefore stem from species differences in the bridging function caused by differences in HSPG-mediated cell surface binding (Peterson et al. 1986; Sanan et al. 1997; Dichek et al. 1998). In normal, *WT*, mice, the preponderance of HL in circulation should increase its access to lipoprotein substrates enriched in triglyceride and phospholipid, generating FFAs for energy and storage. By comparison, lipoprotein substrates for liver-bound HL (including remnants and IDL) would already have been hydrolyzed by lipoprotein lipase (LPL) while passing through muscle and adipose tissue and arrive at the liver with reduced triglyceride (Kersten 2014; Olivecrona and Bengtsson-Olivecrona 1993). Thus, it is possible that the circulating HL in WT mice more effectively mobilizes fatty acids for reesterification and storage than liver-bound HL in hHL-expressing mice, and additional studies are warranted to test this hypothesis.

Because weight gain reflects an imbalance between food intake (energy supply) and energy expenditure (U.S. Department of Health and Human Services PHS 2001), we measured these parameters to further investigate the cause of HL-mediated weight gain. Our finding that HL catalytic function is associated with significantly reduced energy expenditure identifies one potential mechanism to explain the increased weight gain. Conversely, the increased energy expenditure in mice without HL catalytic function explains why they are protected against excessive weight gain. We suspect that increased food intake in hHL-expressing mice, attributable to the catalytic function, although modest, also contributes over time to the increased weight gain in these mice. Additional studies are needed to determine how changes of HL catalytic activity favor positive energy balance.

Also, we found that in mice with rescue of HL catalytic function, lipid oxidation was reduced (as reflected by increased RQ), which suggests that (1) HL activity favors whole body triglyceride storage over lipolysis and FFA oxidation, and that (2) increased adipocyte size and fat mass observed in *hl*^−/−^ mice rescued with hHL may be causally linked to reduced fat oxidation in keeping with our findings in *WT* mice with physiologic levels of murine HL (Chiu et al. 2010). Finally, the modest reduction in ambulatory activity level associated with HL expression, and as seen previously in WT mice, may also favor weight gain in *hl*^−/−^ *hHL* mice by reducing energy demands. Altogether, results from our current study further supports the idea that HL catalytic function rescues the lean phenotype of *hl*^−/−^ mice and extends this property to the human HL enzyme.

Our current results extend our earlier findings of increased weight gain, food intake, percent body fat, adipocyte size and reduced energy expenditure in *WT* mice (with murine HL) to *hl*^−/−^ *hHL* mice with physiologic levels of human HL (Table3) and (Chiu et al. 2010).

Weight gain and obesity lead to serious metabolic derangements including impaired glucose tolerance, insulin resistance and diabetes (Centers-for-Disease-Control 2011). In the current study, the catalytic function was associated with a gender-specific, modestly impaired glucose tolerance in female mice only. The limited effect on glucose homeostasis despite unambiguous differences in body weight and fat mass was also evident in a previous study in *WT* and *hl^−/−^* mice exposed to a HFD (Chiu et al. 2010).

An alternative explanation for the lean phenotype in HL-deficient mice might be reduced weight gain due to adrenal insufficiency. Previous reports suggest that HL may play a role in steroidogenesis, based both on its role in lipoprotein metabolism and its additional localization in adrenal and gonadal tissues (Jansen and Hülsmann 1980; Jansen and Birkenhager 1981; Jansen and De Greef 1981; Dichek et al. 1998, 2006). In support of a role in adrenal steroidogenesis HL-deficiency reduced the corticosterone response to chronic stress in *hl*^−/−^ mice (Dichek et al. 2006). Also, in support of a role in gonadal steroidogenesis, HL-deficiency has been linked to reduced progesterone production and decreased litter size in *hl*^−/−^ mice (Wade et al. 2002). In our current study, we tested the hypothesis that lack of rescue of the lean phenotype in *hl*^−/−^ mice by ciHL is caused by adrenal insufficiency. However, our results demonstrate robust and indistinguishable adrenal corticosterone responses among the three genotypes thus refuting our hypothesis and reducing the likelihood that subclinical adrenal insufficiency contributes to the lack of rescue of the lean phenotype in *hl*^−/−^ *ciHL* mice.

Our principal finding is that HL's catalytic function is required for weight gain. Additionally, we report that the catalytic function mediates this weight gain via multiple mechanisms including reduced energy expenditure, increased food intake, reduced fat oxidation and increased adipocyte size. Although the specific pathways through which these effects occur remain unknown, we speculate that one of these involves the effect of HL activity to increase lipoprotein hydrolysis and hence FFA generation, and favoring diversion to fat deposition rather than oxidation. As obesity now affects more than 400 million people (Nguyen and El-Serag 2010) and predisposes to diabetes (Centers-for-Disease-Control 2011) and cardiovascular disease (Eckel and Krauss 1998), chronic conditions with high morbidity and mortality, the need is acute for novel obesity treatments. Our data suggests that inhibition of hepatic lipase catalytic activity could be investigated as a new approach to obesity treatment.

## References

[b1] Beigneux AP, Davies BS, Gin P, Weinstein MM, Farber E, Qiao X (2007). Glycosylphosphatidylinositol-anchored high-density lipoprotein-binding protein 1 plays a critical role in the lipolytic processing of chylomicrons. Cell Metab.

[b2] Brunzell JD, Deeb SS, Scriver CR, Beaudet AL, Sly WS, Valle D, Childs B, Kinzler KW, Vogelstein B (2001). Familial lipoprotein lipase deficiency, apoC-II deficiency, and hepatic lipase deficiency. The metabolic and molecular bases of inherited disease.

[b3] Carr MC, Hokanson JE, Deeb SS, Purnell JQ, Mitchell ES, Brunzell J (1999). A hepatic lipase gene promoter polymorphism attenuates the increase in hepatic lipase activity with increasing intra-abdominal fat in women. Arterioscler. Thromb. Vasc. Biol.

[b4] Carr MC, Hokanson JE, Zambon A, Deeb SS, Barrett PHR, Purnell JQ (2001). The contribution of intra-abdominal fat to gender differences in hepatic lipase activity and low/high density lipoprotein heterogeneity. J. Clin. Endocrinol. Metab.

[b5] Centers-for-Disease-Control (2011). http://www.cdc.gov/diabetes/pubs/pdf/ndfs_2011.pdf.

[b6] Chiu HK, Qian K, Ogimoto K, Morton GJ, Wisse BE, Agrawal N (2010). Mice lacking hepatic lipase are lean and protected against diet-induced obesity and hepatic steatosis. Endocrinology.

[b7] Connelly PW (1999). The role of hepatic lipase in lipoprotein metabolism. Clin. Chim. Acta.

[b8] Davies BSJ, Beigneux AP, Tu RH, Barnes Y, Gin P, Weinstein MM (2010). GPIHBP1 is responsible for the entry of lipoprotein lipase into capillaries. Cell Metab.

[b9] Despres JP, Ferland M, Moorjani S, Nadeau A, Tremblay A, Lupien PJ (1989). Role of hepatic-triglyceride lipase activity in the association between intra-abdominal fat and plasma HDL cholesterol in obese women. Arteriosclerosis.

[b10] Diard P, Malewiak M-I, Lagrange D, Griglio S (1994). Hepatic lipase may act as a ligand in the uptake of artificial chylomicron remnant-like particles by isolated rat hepatocytes. Biochem. J.

[b11] Dichek HL, Brecht W, Fan J, Ji Z-S, McCormick SPA, Akeefe H (1998). Overexpression of hepatic lipase in transgenic mice decreases apolipoprotein B-containing and high density lipoproteins. J. Biol. Chem.

[b12] Dichek HL, Johnson SM, Akeefe H, Lo GT, Sage E, Yap CE (2001). Hepatic lipase overexpression lowers remnant and LDL levels by a noncatalytic mechanism in LDL receptor-deficient mice. J. Lipid Res.

[b13] Dichek HL, Qian K, Agrawal N (2004a). The bridging function of hepatic lipase clears plasma cholesterol in LDL receptor-deficient “apoB-48-only” and “apoB-100-only” mice. J. Lipid Res.

[b14] Dichek HL, Qian K, Agrawal N (2004b). Divergent effects of the catalytic and bridging functions of hepatic lipase on atherosclerosis. Arterioscler. Thromb. Vasc. Biol.

[b15] Dichek HL, Agrawal N, El Andaloussi N, Qian K (2006). Attenuated corticosterone response to chronic ACTH stimulation in hepatic lipase-deficient mice: evidence for a role for hepatic lipase in adrenal physiology. Am. J. Physiol. Endocrinol. Metab.

[b16] Eckel RM, Krauss RM (1998). American Heart Association Call to Action: obesity as a major risk factor for coronary heart disease. AHA Nutrition Committee. Circulation.

[b17] Fan J, Wang J, Bensadoun A, Lauer SJ, Dang Q, Mahley RW (1994). Overexpression of hepatic lipase in transgenic rabbits leads to a marked reduction of plasma high density lipoproteins and intermediate density lipoproteins. Proc. Natl Acad. Sci. USA.

[b18] Gelling RW, Yan W, Al-Noori S, Pardini A, Morton GJ, Ogimoto K (2008). Deficiency of TNFalpha converting enzyme (TACE/ADAM17) causes a lean, hypermetabolic phenotype in mice. Endocrinology.

[b19] Homanics GE, de Silva HV, Osada J, Zhang SH, Wong H, Borensztajn J (1995). Mild dyslipidemia in mice following targeted inactivation of the hepatic lipase gene. J. Biol. Chem.

[b20] Iverius P-H, Brunzell JD (1985). Human adipose tissue lipoprotein lipase: Changes with feeding and relation to postheparin plasma enzyme. Am. J. Physiol.

[b21] Jackson RL, Boyer PD (1983). Lipoprotein lipase and hepatic lipase. The enzymes.

[b22] Jansen H, Birkenhager JC (1981). Liver lipase-like activity in human and hamster adrenocortical tissue. Metabolism.

[b23] Jansen H, De Greef WJ (1981). Heparin-releasable lipase activity of rat adrenals, ovaries and testes. Biochem. J.

[b24] Jansen H, Hülsmann WC (1980). Heparin-releasable (liver) lipase(s) may play a role in the uptake of cholesterol by steroid-secreting tissues. Trends Biochem. Sci.

[b25] Jansen H, Kalkman C, Zonneveld AJ, Hulsmann WC (1979). Secretion of triacylglycerol hydrolase activity by isolated parenchymal rat liver cells. FEBS Lett.

[b26] Ji Z-S, Lauer SJ, Fazio S, Bensadoun A, Taylor JM, Mahley RW (1994). Enhanced binding and uptake of remnant lipoproteins by hepatic lipase-secreting hepatoma cells in culture. J. Biol. Chem.

[b27] Ji Z-S, Dichek HL, Miranda RD, Mahley RW (1997). Heparan sulfate proteoglycans participate in hepatic lipase- and apolipoprotein E-mediated binding and uptake of plasma lipoproteins, including high density lipoproteins. J. Biol. Chem.

[b28] Kaiyala KJ, Schwartz MW (2011). Toward a more complete (and less controversial) understanding of energy expenditure and its role in obesity pathogenesis. Diabetes.

[b29] Kaiyala KJ, Morton GJ, Leroux BG, Ogimoto K, Wisse B, Schwartz MW (2010). Identification of body fat mass as a major determinant of metabolic rate in mice. Diabetes.

[b30] Kersten S (2014). Physiological regulation of lipoprotein lipase. Biochim. Biophys. Acta.

[b31] Kounnas MZ, Chappell DA, Wong H, Argraves WS, Strickland DK (1995). The cellular internalization and degradation of hepatic lipase is mediated by low density lipoprotein receptor-related protein and requires cell surface proteoglycans. J. Biol. Chem.

[b32] Kuusi T, Kinnunen PKJ, Nikkilä EA (1979). Hepatic endothelial lipase antiserum influences rat plasma low and high density lipoproteins in vivo. FEBS Lett.

[b33] Kuusi T, Saarinen P, Nikkilä EA (1980). Evidence for the role of hepatic endothelial lipase in the metabolism of plasma high density lipoprotein2 in man. Atherosclerosis.

[b34] Mehrabian M, Wen PZ, Fisler J, Davis RC, Lusis AJ (1998). Genetic loci controlling body fat, lipoprotein metabolism, and insulin levels in a multifactorial mouse model. J. Clin. Invest.

[b35] Morton GJ, Kaiyala KJ, Fisher JD, Ogimoto K, Schwartz MW, Wisse BE (2011). Identification of a physiological role for leptin in the regulation of ambulatory activity and wheel running in mice. Am. J. Physiol. Endocrinol. Metab.

[b36] Nguyen DM, El-Serag HB (2010). The epidemiology of obesity. Gastroenterol. Clin. North Am.

[b37] Nilsson-Ehle P, Borensztajn J (1987). Measurements of lipoprotein lipase activity. Lipoprotein lipase.

[b38] Olivecrona T, Bengtsson-Olivecrona G (1993). Lipoprotein lipase and hepatic lipase. Curr. Opin. Lipidol.

[b39] Peterson J, Bengtsson-Olivecrona G, Olivecrona T (1986). Mouse preheparin plasma contains high levels of hepatic lipase with low affinity for heparin. Biochim. Biophys. Acta.

[b40] Purnell JQ, Kahn SE, Albers JJ, Nevin DN, Brunzell JD, Schwartz RS (2000). Effect of weight loss with reduction of intra-abdominal fat on lipid metabolism in older men. J. Clin. Endocrinol. Metab.

[b41] Purves RD (1992). Optimum numerical integration methods for estimation of area-under-the-curve (AUC) and area -under-the-moment-curve (AUMC). J. Pharmacokinet. Biopharm.

[b42] Qian K, Agrawal N, Dichek HL (2007). Reduced atherosclerosis in chow-fed mice expressing high levels of a catalytically inactive human hepatic lipase. Atherosclerosis.

[b43] Sanan DA, Fan J, Bensadoun A, Taylor JM (1997). Hepatic lipase is abundant on both hepatocyte and endothelial cell surfaces in the liver. J. Lipid Res.

[b44] Sendak RA, Berryman DE, Gellman G, Melford K, Bensadoun A (2000). Binding of hepatic lipase to heparin: identification of specific heparin-binding residues in two distinct positive charge clusters. J. Lipid Res.

[b45] van Tilbeurgh H ARoussel, Lalouel J-M, Cambillau C (1994). Lipoprotein lipase. Molecular model based on the pancreatic lipase x-ray structure: Consequences for heparin binding and catalysis. J. Biol. Chem.

[b46] U.S. Department of Health and Human Services PHS (2001).

[b47] Wade RL, Van Andel RA, Rice SG, Banka CL, Dyer CA (2002). Hepatic lipase deficiency Attenuates Mouse Ovarian Progesterone Production Leading to Decreased Ovulation and Reduced Litter Size. Biol. Reprod.

[b48] Warden CH, Fisler JS, Shoemaker SM, Wen P-Z, Svenson KL, Pace MJ (1995). Identification of four chromosomal loci determining obesity in a multifactorial mouse model. J. Clin. Invest.

